# The Impact of Advanced Care Planning on Hospice Utilization in Patients with Cancer: A Nationwide Analysis in Korea

**DOI:** 10.3390/cancers17091471

**Published:** 2025-04-27

**Authors:** Woorim Kim, Boram Kim, Minju Kim, Jin Young Choi

**Affiliations:** 1 National Hospice Center, National Cancer Control Institute, National Cancer Center, Goyang 10408, Republic of Korea; 2 Department of Hospice and Palliative Service, National Cancer Center, Goyang 10408, Republic of Korea

**Keywords:** hospice, palliative care, advance care planning, life-sustaining treatment, cancer deaths

## Abstract

**Background:** Hospice and palliative care play key roles in facilitating goals-of-care discussions and managing symptoms in end-of-life (EOL) care for cancer patients. In this study, the association between completion of advance care planning (ACP), which refers to decisions regarding life-sustaining treatment, and hospice utilization in patients with cancer is examined. **Methods:** Data from the Korea-Clinical Data Utilization Network for Research Excellence were used. The study population included individuals diagnosed with one of the five leading causes of cancer-related deaths in South Korea. The association between the completion of ACP and hospice utilization was analyzed using multivariable logistic regression analysis. **Results:** The study population included 50,574 patients with terminal cancer, of whom 10,530 (20.8%) had received hospice care. Self-determined ACP was completed by 9271 (18.3%) participants and non-self-determined ACP by 8654 (17.1%). Patients with ACP were more likely to receive hospice care, with the self-determined group (OR, 5.46; 95% CI: 5.13–5.81) showing a stronger association than the non-self-determined group (OR, 1.27; 95% CI: 1.18–1.37). **Conclusions:** Patients with cancer who completed ACP were more likely to use hospice care services. These findings indicate the potential importance of ACP decisions in providing EOL care.

## Simple Summary

Hospice and palliative care help patients with cancer to engage in discussions about their goals related to their care and to live more comfortably at their end of life. Advance care planning (ACP) involves making decisions about patients’ future medical treatment, including whether to receive life-sustaining treatments. This study examined the relationship between ACP completion and hospice care use among people with cancer in South Korea at their end of life using a large, national dataset. We found that patients who made their own care decisions in advance were significantly more likely to use hospice services. Hospice use also increased when decisions were made by family members on behalf of patients who lost their decision-making capacity. These findings highlight the potential role of ACP in ensuring that patients receive appropriate care and support as they approach their end of life.

## 1. Introduction

Cancer patients and their families often face difficulties in making end-of-life (EOL) care decisions, highlighting the critical role of clinicians in developing appropriate treatment plans for terminally ill patients. Hospice and palliative care play key roles in facilitating goals-of-care discussions and managing symptoms in such patients; furthermore, palliative care has been demonstrated to enhance the quality of life of terminally ill cancer patients while reducing emergency room visits, hospitalizations, and admissions to intensive care units [1,2]. Accordingly, both the American Society of Clinical Oncology and the National Comprehensive Cancer Network recommend that institutions establish processes for integrating palliative care into standard cancer care and that healthcare professionals engage in discussions about palliative care services for patients diagnosed with advanced cancer [3,4]. Nevertheless, many cancer patients receive aggressive medical interventions at the EOL, with a relatively short duration between hospice enrollment and death [5]. Among the various factors influencing the use of hospice care services and preferences regarding life-sustaining treatment (LST), a key factor is the challenge for healthcare professionals and patients to openly discuss these topics [6,7]. While seriously ill cancer patients are generally open to such discussions, clinicians still encounter difficulties in effectively conveying prognostic information, clarifying patient concerns and preferences, and engaging in future planning, even when strong clinician–patient relationships are established [7].

Hospice care was introduced in Korea in the 1960s, and inpatient hospice services have been officially covered by the National Health Insurance (NHI) system since 2015 [8,9]. Following this development, hospice care has expanded to include various types of services, such as home-based services. Patients can access these services through medical institutions certified by the Ministry of Health and Welfare. Hospice care is provided to terminally ill patients with cancer and other chronic diseases, with the majority being patients with terminal cancer. Hospice use in Korea is growing gradually but remains limited. Approximately one in five cancer patients utilize hospice services [8], resulting in a lower level of uptake compared to other countries [10].

Advance care planning (ACP) is the process of discussing and documenting a patient’s preferences for future medical treatment and goals of care in the event of the loss of one’s decision-making capacity [11]. Previous studies have shown that ACP can enhance communication between patients and healthcare professionals, increase patients’ awareness of their disease and prognosis, and promote patient-centered care aligned with their goals [11,12]. In South Korea, although do-not-resuscitate orders had previously been used in clinical practice, there was no formal legal framework to address decisions to withhold or withdraw LST. This situation gave rise to legal debates and public discourse regarding such decisions, prompting the development of legislation to support patient-centered care planning in anticipation of future loss of decision-making capacity [13]. This led to the enactment of the Act on Decisions on Life-Sustaining Treatment, which came into effect in 2018. According to this law, patients in Korea are legally able to document their ACP. They can express their EOL preferences by completing an advance directive (AD), which may later be reviewed and acknowledged by their physician, or by having their wishes reflected in a physician order for life-sustaining treatment (POLST). Following the enactment of the law, the number of patients completing ACP increased, and decisions were made at an earlier stage of illness progression [14]. In many Asian societies, treatment preferences are not based solely on individual autonomy but are shaped through familial relationships and responsibilities [15,16]. This cultural context is reflected in the way ACP is practiced across Asia and has shaped the legal framework for end-of-life decision-making in Korea. There is no legal provision that explicitly allows patients to designate their surrogate decision-makers. Instead, if patients lose their decision-making capacity owing to advanced illness and have not completed any documents, their family members may act as default surrogates in Korea [13,17]. As the legal framework is still in its early phase, ACP in Korea shows low uptake, with substantial involvement of family members [17].

Growing evidence suggests that ACP is associated with less aggressive treatment and better EOL care. However, evidence regarding its association with improved hospice utilization among terminally ill cancer patients is lacking. Previous studies primarily examined the impact of ACP on hospice utilization among older adults, nursing home residents, and patients with various medical conditions [11,18]. Even when studies have been conducted on advanced cancer patients, they have been limited to specific cancer types or have focused on the relationship between hospice utilization and discussions regarding EOL care rather than on completed ACPs in written form [19,20,21]. Therefore, the association between ACP completion and hospice utilization among patients with cancer in the EOL phase was examined in this study.

## 2. Methods

### 2.1. Data and Study Population

This study used the Cancer Public Library Database provided by the Korea-Clinical Data Utilization Network for Research Excellence (K-CURE), a comprehensive population-based database that integrates data from multiple national sources, including the Korea Central Cancer Registry, National Health Insurance Service, Health Insurance Review and Assessment Service, and Statistics Korea [22]. The study population included patients who were first diagnosed with cancer between 2018 and 2021 and died within one year of diagnosis. Patients who were diagnosed before the implementation of the Act on Decisions on Life-Sustaining Treatment were excluded. Additionally, individuals with missing data on key explanatory variables, including sex, age, major cancer type, and summary stage, were excluded from the analysis.

In this study, the impact of ACP on hospice utilization among patients with cancer is examined. The study population consisted of individuals diagnosed with cancer, specifically those whose cancer type ranked among the top five leading causes of cancer-related deaths (stomach, colorectal, liver, pancreas, and lung) in Korea during the study period [23], according to the International Classification of Diseases, 10th Revision. A total of 106,551 patients were recorded as being diagnosed with cancer and died within one year of diagnosis in the data used. Of these individuals, 50,574 individuals had stomach, colorectal, liver, pancreas, or lung cancer, which comprised the study population.

### 2.2. Variables

The outcome variable in this study was hospice utilization status. Hospice utilization was defined as the use of inpatient or home-based services, identified based on National Health Insurance (NHI) claims data. This was because these two types of services are both offered and reimbursed under the NHI in Korea, in which patients are required to pay 5 to 10 percent of total incurred costs as out-of-pocket payment. Inpatient hospice provides an array of services, such as pain and symptom management, psychological consultations, and therapeutic programs in an inpatient setting, whereas home-based services provide such interventions at home by a care team from a certified hospital. Individuals were categorized based on whether they utilized hospice services (yes) or did not utilize them (no).

The primary independent variable was ACP, which refers to decisions regarding LSTs. We categorized ACP into two groups based on the method of documentation and the extent of patient involvement. Cases were classified as self-determined when ACP was completed by the patient through either an AD or a POLST. In contrast, cases were considered not self-determined when ACP was completed after the patient had lost decision-making capacity, and decisions were made either based on family members’ statements regarding the patient’s previously expressed wishes or through surrogate decision-making by family members in the absence of such statements. These were identified based on the NHI claims code (WU110, WU210, WU310, WU410, WU120, WU220, WU320, WU420, WU130, WU230, WU330, WU430, IA711, IA712, IA713, IA714, IA721, IA722, IA723, IA724, IA731, IA732, IA733, IA734) used for reimbursement. Only ACP made before hospice utilization was incorporated in the analysis as this study aimed to investigate whether prior ACP leads to a difference in the utilization of hospice services.

In addition to the primary independent variables, various covariates were included in the analysis. The sociodemographic variables included sex, age, income level, region, healthcare service type, and hospital type. Age was categorized into 10-year intervals (20–29, 30–39, 40–49, 50–59, 60–69, 70–79, and ≥80 years old). Household income levels were classified into four groups based on the deciles of NHI premiums. All individuals are covered by the NHI or Medical Aid in Korea, in which the level of NHI premium for each individual is set based on household income and assets. As such, the level of NHI premium was used as a proxy for income level. The regions were categorized into four groups based on the administrative regions of Korea: the capital city (Seoul), the capital area (Gyeonggi and Incheon), metropolitan cities (Busan, Daegu, Gwangju, Daejeon, and Ulsan), and other provinces. Healthcare service type was determined based on at least one claim made within the last year before death. Patients were categorized as either inpatients or outpatients, according to their last recorded healthcare service. Healthcare service utilization was further classified by the type of institution (tertiary hospitals, general hospitals, hospitals, and clinics). Clinical variables included the surveillance, epidemiology, and end results summary stage at the initial cancer diagnosis (localized, regional, distant, or unknown), and major cancer types. The major cancer types were lung, stomach, colorectal, liver, and pancreatic cancers. Details of the variables used in this study have been previously described [22].

### 2.3. Statistical Analysis

Descriptive statistics were used to examine the general characteristics of the study population according to hospice utilization and ACP status. Categorical variables are presented as frequencies and percentages, and group comparisons were performed using the chi-square test. To evaluate the association between hospice utilization and ACP status, a multivariable logistic regression analysis was conducted. The dependent variable was hospice utilization (yes/no), whereas the independent variables were ACP status, sociodemographic factors, and clinical characteristics. Adjusted odds ratios (ORs) and 95% confidence intervals (CIs) were determined. Statistical significance was set at *p* < 0.05. All statistical analyses were performed using the SAS software (version 9.4; SAS Institute, Cary, NC, USA).

## 3. Results

The general characteristics of the study population are summarized in Table 1. The study population comprised 50,574 patients with terminal cancer, 10,530 (20.8%) of whom received hospice care.

The results of the analysis of the relationship between hospice utilization and advanced are presented in Table 2. Patients with ACP were more likely to receive hospice care, with the self-determined group (OR, 5.46; 95% CI: 5.13–5.81) showing a stronger association than the non-self-determined group (OR, 1.27; 95% CI: 1.18–1.37). Different factors were also related to the use of hospital services, including age, where individuals aged between 30 to 69 years were less likely to use ACP than those aged 80 years or above. A higher income level was also positively associated with hospice utilization (OR, 1.25; 95% CI: 1.17–1.34). Regional differences were observed as individuals living in metropolitan cities (OR, 1.80; 95% CI: 1.66–1.95) and the capital area (OR, 1.17; 95% CI: 1.08–1.26) had a higher likelihood of using hospice services than those in the capital city. Regarding the type of cancer, compared to lung cancer, pancreatic cancer showed a significant association with hospice utilization. A more advanced cancer stage was also related to higher hospice utilization as distant metastasis (OR, 2.12; 95% CI: 1.94–2.31) showed an association.

## 4. Discussion

According to this study, a substantial proportion of patients with terminal cancer did not receive hospice care during their last year of life, which is associated with multiple factors. Notably, a significant association was found between ACP completion and increased hospice care utilization. These findings have important implications for understanding the factors associated with hospice utilization in patients with terminal cancer and advance existing research by providing robust evidence on the association between ACP completion and hospice use based on a large, nationwide database. Specifically, standardized population-based cancer data sourced from government and public agencies, encompassing cancer registries, insurance claims, and mortality data, were used. This approach addresses the limitations of previous studies that relied on hospital-based or regional data [19,20,24], which often lacked a comprehensive inclusion of patients with various types of cancer or had a limited number of study participants. Furthermore, the ACP was identified using insurance claims data in this study, accurately reflecting actual hospice utilization and ACP registration, rather than being inferred from medical records or patient-family reports. Previous studies have highlighted the inconsistencies in the reporting of EOL communication. This study found discrepancies between caregiver and physician reports [25], whereas another study demonstrated that the definition of EOL discussions, based on medical records or patient/surrogate reports, led to different associations with EOL care outcomes [26]. Consequently, because this study analyzed nationally representative data with reduced information bias, its findings can be broadly generalized to patients with terminal cancer. This study is also distinguished from previous research by its comprehensive inclusion of AD, POLST, and surrogate decision-making within the scope of the ACP, and comparisons were made based on who the decision-maker was. Finally, this study examined ACP and hospice use among patients with terminal cancer diagnosed following the introduction of legally recognized and registered ACP in Korea’s healthcare system.

Prior studies that assessed hospice use found significant associations with EOL discussions, defined as conversations with physicians about EOL care, goals of care, treatment preferences, life expectancy, AD, POLST, and a combination of these topics [20,26]. These discussions have also been associated with less aggressive medical care near death and earlier hospice referrals among patients [21,26], further supporting our findings. ACP has been shown to improve prognostic awareness and increase our understanding of future treatment options for cancer patients [12]. Furthermore, it facilitates the communication of EOL care preferences, enabling healthcare providers to respect their wishes [5]. However, a previous study showed that cancer patients who lack a sense of connection with their healthcare providers experience challenges in communication and shared decision-making, and often feel uninformed about treatment options [27]. This may contribute to the limited understanding and low utilization of hospice care among patients with terminal cancer, underscoring the importance of proactive EOL discussions regarding healthcare provider care preferences. Our study also found that self-determined ACP was more strongly associated with hospice use, whereas non-self-determined ACP was positively associated. Given that late EOL discussions have been associated with more aggressive care and shorter hospice stays [26], surrogate decision-making may be related to time limitations in hospice care decisions. Surrogates often have difficulty in accurately guessing patients’ EOL treatment preferences, and neither patient designation nor prior discussions have been shown to significantly improve predictive accuracy [28]. This is further supported by previous studies showing that patients whose end-of-life decisions were made by family members were more likely to receive aggressive care, including higher rates of admission to intensive care units and visits to emergency departments, compared to those who had completed ACP directly [17,29]. One possible explanation is that such tendencies are influenced by factors that shape family decision-making, including a sense of filial duty, a belief in doing everything possible, and a form of caregivers’ obsession with pursuing aggressive treatments despite poor prognosis [29]. These findings highlight the need for healthcare providers to engage in early ACP discussions directly with terminally ill cancer patients to clarify their preferences regarding future treatment, including hospice care. Furthermore, cultivating a cultural environment where conversations about death can take place more comfortably may contribute to more appropriate EOL care decisions grounded in patient autonomy.

Previous studies have reported low utilization of hospice care among terminally ill patients [30,31], which is consistent with our findings. Despite the incurable nature of the illness, a significant number of terminal cancer patients continue to receive aggressive medical care at the end of their lives [5,32,33,34]. Hospice care is often initiated near the end of life and the interval between hospice enrollment and death is short [5,35]. Previous studies have found that patients with terminal cancer favor high-quality, comfort-focused care over LST [36,37]. Engagement in EOL discussions with physicians was associated with greater concordance between patient preferences and care received [36], underscoring the essential role of healthcare providers in guiding care decisions for patients with terminal cancer. This is further supported by previous studies demonstrating that healthcare provider recommendations and consultations regarding hospice enrollment significantly increase the likelihood of hospice utilization [5,32]. Further research is needed to develop and evaluate effective strategies aimed at increasing hospice care utilization among patients with terminal cancer.

Consistent with previous findings [6,30,38], according to this study, female patients and those with higher income levels were more likely to utilize hospice care. Gender differences in hospice utilization may result from men’s tendency to decline supportive services or sex-specific variations in symptom presentation and prognosis [30]. Our finding that higher-income groups are more likely to utilize hospice services is consistent with a previous study [39], possibly reflecting their greater acceptance of the withdrawal of futile life-sustaining treatments and a stronger preference for active pain control [40]. This study also revealed regional variations in hospice utilization, potentially reflecting disparities in hospice availability and timely access reported in prior research [6]. Supporting this observation, the proportion of newly registered patients who utilized hospice care institutions within their own region in 2021 varied widely across Korea. This rate ranged from approximately 28% to 97%, with higher levels observed in the capital area and metropolitan cities compared to the capital city and other provinces [8]. These patterns are consistent with prior research in South Korea, which suggests that regional differences in EOL care preferences may partly reflect structural disparities in access to information and perceived availability of healthcare services, particularly between metropolitan and non-metropolitan areas [41]. Hospice utilization is closely linked to inpatient- and home-based services and reflects the structural characteristics of the Korean healthcare system. For most of the study period, **primarily inpatient hospice services were** eligible for NHI reimbursement. Although the NHI initiated the coverage of home-based hospice services in late 2020, the number of claims for this service during the study period was very limited. Notably, many clinics offer inpatient hospice care, unlike routine oncological care where such services are rarely available in clinical settings [8].

This study had several limitations. First, there may have been relevant cases that were not considered ACP in this study. Research data did not include cases where ACP was documented but no insurance claim was submitted or where legally unrecognized documents, such as do-not-resuscitate orders, were completed. Additionally, several variables [8,9] previously associated with hospice utilization among cancer patients were not assessed because they were not included in the database used in this study, despite their established relevance. These variables included patient-related factors (e.g., performance status and awareness of hospice/palliative care), provider-related factors (e.g., knowledge about hospice/palliative care and prognostication challenges), and healthcare-related factors (e.g., access to hospice/palliative care) [38,42]. Last, the analysis was limited to patients with cancer who died within one year of diagnosis as it aimed to investigate the relationship between ACP and hospice use in terminally ill patients. This definition was also applied considering that only patients who were diagnosed as being terminally ill, generally projected as having less than six months of life, in Korea were included. Still, the results should be interpreted taking into account that longer term survivors, who may have different patterns of ACP completion and hospice utilization, were excluded from the analysis. Despite such limitations, this study is unique in that it examined the association between ACP completion and hospice utilization in patients with cancer using longitudinal data. This allowed the revelation of a temporal relationship between ACP completion and hospice care utilization.

## 5. Conclusions

In this study, the association between ACP completion and hospice utilization among terminally ill patients with cancer was examined. These findings suggest that patients who completed ACP were more likely to use hospice services, a relationship that was consistently observed across various factors. These results emphasize the critical role of healthcare providers in initiating and facilitating ACP discussions to improve the quality of EOL care. Further research is needed to investigate how ACP discussions promote timely hospice enrollment and identify strategies for healthcare providers to effectively utilize ACP to enhance hospice use.

## Figures and Tables

**Table 1:** General characteristics of the study population.

	Total	Hospice Utilization				*p*-Value
		No		Yes		
		N	%	N	%	
Advance care planning						
No	32,649	27,087	67.6	5562	52.8	<0.0001
Yes (Not self-determined)	8654	7462	18.6	1192	11.3	
Yes (Self-determined)	9271	5495	13.7	3776	35.9	
Sex						
Male	35,596	28,805	71.9	6791	64.5	<0.0001
Female	14,978	11,239	28.1	3739	35.5	
Age						
20–29	72	54	0.1	18	0.2	<0.0001
30–39	460	369	0.9	91	0.9	
40–49	1959	1588	4.0	371	3.5	
50–59	6322	5168	12.9	1154	11.0	
60–69	10,951	8824	22.0	2127	20.2	
70–79	16,713	13,123	32.8	3590	34.1	
80+	14,097	10,918	27.3	3179	30.2	
Income level						
Low	12,319	9867	24.6	2452	23.3	<0.0001
Low–middle	13,702	10,990	27.4	2712	25.8	
Middle–high	9545	7605	19.0	1940	18.4	
High	15,008	11,582	28.9	3426	32.5	
Region						
Capital city	8598	6882	17.2	1716	16.3	<0.0001
Capital area	12,650	9896	24.7	2754	26.2	
Metropolitan cities	9589	7208	18.0	2381	22.6	
Other provinces	19,737	16,058	40.1	3679	34.9	
Healthcare service						
Outpatient	6004	5678	14.2	326	3.1	<0.0001
Inpatient	44,570	34,366	85.8	10,204	96.9	
Hospital type						
Tertiary hospital	20,211	17,972	44.9	2239	21.3	<0.0001
General hospital	23,680	18,291	45.7	5389	51.2	
Hospital	4317	2739	6.8	1578	15.0	
Clinic	2366	1042	2.6	1324	12.6	
Cancer type						
Stomach	7445	5789	14.5	1656	15.7	<0.0001
Colorectal	6174	4905	12.2	1269	12.1	
Liver	10,586	8545	21.3	2041	19.4	
Pancreatic	7658	5275	13.2	2383	22.6	
Lung	18,711	15,530	38.8	3181	30.2	
Cancer stage						
Localized	6004	5176	12.9	828	7.9	<0.0001
Regional	11,622	9643	24.1	1979	18.8	
Distant	27,349	20,699	51.7	6650	63.2	
Unknown	5599	4526	11.3	1073	10.2	
Total	50,574	40,044	79.2	10,530	20.8	

**Table 2:** The association between hospice utilization and advance care planning status.

	Odds Ratio	95% Confidence Interval
Advance care planning		
No	1.00	
Yes (Not self-determined)	1.27	(1.18–1.37)
Yes (Self-determined)	5.46	(5.13–5.81)
Sex		
Male	1.00	
Female	1.19	(1.13–1.26)
Age		
20–29	1.02	(0.55–1.87)
30–39	0.66	(0.51–0.86)
40–49	0.62	(0.54–0.71)
50–59	0.61	(0.55–0.66)
60–69	0.74	(0.69–0.80)
70–79	0.93	(0.87–0.99)
80+	1.00	
Income level		
Low	1.00	
Low–middle	1.06	(0.99–1.14)
Middle–high	1.11	(1.03–1.20)
High	1.25	(1.17–1.34)
Region		
Capital city	1.00	
Capital area	1.17	(1.08–1.26)
Metropolitan cities	1.80	(1.66–1.95)
Other provinces	1.01	(0.94–1.09)
Healthcare service		
Outpatient	1.00	
Inpatient	11.56	(9.97–13.39)
Hospital type		
Tertiary hospital	1.00	
General hospital	3.14	(2.95–3.33)
Hospital	8.75	(8.00–9.57)
Clinic	51.95	(45.37–59.48)
Cancer type		
Lung	1.00	
Stomach	1.48	(1.38–1.60)
Colorectal	1.24	(1.15–1.35)
Liver	1.43	(1.32–1.53)
Pancreatic	2.07	(1.93–2.22)
Cancer stage		
Localized	1.00	
Regional	1.34	(1.22–1.48)
Distant	2.12	(1.94–2.31)
Unknown	1.44	(1.29–1.61)

## Data Availability

The data presented in this study are available from the Cancer Public Library Database, a publicly accessible resource provided by the Korea-Clinical Data Utilization Network for Research Excellence. Data can be accessed after approval at https://k-cure.mohw.go.kr.

## Abbreviations

The following abbreviations are used in this manuscript:

ACP	Advance care planning
EOL	End of life
LST	Life-sustaining treatment
AD	Advance directive
POLST	Physician order for life-sustaining treatment
